# Establishing performance standards for child development: learnings from the ECDI2030

**DOI:** 10.1186/s41043-023-00483-2

**Published:** 2023-12-12

**Authors:** Nicole Petrowski, Filipa de Castro, Susan Davis-Becker, Melissa Gladstone, Claudia Regina Lindgren Alves, Yvonne Becher, Jennifer Grisham, Kirsten Donald, Meta van den Heuvel, Gwendoline Kandawasvika, Shazia Maqbool, Fahmida Tofail, Tao Xin, Pia Zeinoun, Claudia Cappa

**Affiliations:** 1 UNICEF, Data and Analytics Section, 3 UN Plaza, New York, NY 10017 USA; 2 Formerly with UNICEF, Data and Analytics Section, 3 UN Plaza, New York, NY 10017 USA; 3 ACS Ventures, 11035 Lavender Hill Drive #160-433, Las Vegas, NV 89135 USA; 4 Department of Women and Children’s Health, Liverpool School of Tropical Medicine, University of Liverpool, Pembroke Place, Liverpool, L3 5QA UK; 5 Department of Pediatrics, Universidade Federal de Minas Gerais/School of Medicine, Minas Gerais, Brazil; 6 The Child Development Centre, 4/F Prime Mansion, 183-187 Johnston Road, Wan Chai, Hong Kong; 7 Early Childhood Laboratory, University of Kentucky, 621 S. Limestone, Lexington, KY 40506-0657 USA; 8 Division of Developmental Pediatrics, Red Cross War Memorial Children’s Hospital and Neuroscience Institute, University of Cape Town, Cape Town, South Africa; 9 Hospital for Sick Children, 555 University Ave, Toronto, ON M5G 1X8 Canada; 10 Primary Health Sciences Department, Faculty of Medicine and Health Sciences, University of Zimbabwe, Mt Pleasant, P.O. Box MP167, Harare, Zimbabwe; 11 Developmental-Behavioral Pediatrics Department, The Children’s Hospital and Institute of Child Health, Lahore, Pakistan; 12 International Centre for Diarrhoeal Disease Research, GPO Box 128, Dhaka, 1000 Bangladesh; 13 National Assessment Center for Education Quality, Ministry of Education, Beijing, China; 14 Embrace NGO, Beirut, Lebanon

**Keywords:** Child development, Performance standards, Measurement, SDGs

## Abstract

**Background:**

Standards of early childhood development (ECD) are needed to determine whether children living in different contexts are developmentally on track. The Early Childhood Development Index 2030 (ECDI2030) is a population-level measure intended to be used in household surveys to collect globally comparable data on one of the indicators chosen to monitor progress toward target 4.2 of the Sustainable Development Goals: The proportion of children aged 24–59 months who are developmentally on track in health, learning and psychosocial well-being.

**Methods:**

To define performance cut-scores for the ECDI2030 we followed a criterion-referenced standard setting exercise using the modified Angoff method. The exercise gauged the expectations from 15 global experts in ECD and was informed by representative population data collected in Mexico and the State of Palestine. The final calibrated age-specific performance cut-scores were applied to these data to estimate the proportion of children developmentally on track, disaggregated by background characteristics, including the child's sex and attendance to early childhood education.

**Results:**

Through a process of standard setting, we generated robust performance standards for the ECDI2030 by establishing five age-specific cut-scores to identify children as developmentally on track.

**Conclusions:**

This paper demonstrated how the standard setting methodology, typically applied to measures in the health and education fields, could be applied to a measure of child development. By creating robust criterion-referenced standards, we have been able to ensure that the cut-scores related to age for the ECDI2030 are based on performance standards set by global experts in the ECD field for defining on and off track development.

**Supplementary Information:**

The online version contains supplementary material available at 10.1186/s41043-023-00483-2.

## Background

In September 2015, the United Nations General Assembly adopted the 2030 Agenda for Sustainable Development, an ambitious plan of action for people, planet and prosperity [15]. The Agenda is comprised of 17 Sustainable Development Goals (SDGs), 169 targets and over 200 indicators. Early childhood development (ECD) is a necessary and central component of this agenda and is ackowledged as such through the inclusion of a dedicated target (4.2) within these SDGs. Indicator 4.2.1 has been chosen to monitor progress on ECD by measuring: The proportion of children aged 24–59 months who are developmentally on track in health, learning and psychosocial well-being.

As the custodian agency for indicator 4.2.1, the United Nations Children’s Fund (UNICEF) led methodological work to design the Early Childhood Development Index 2030 (ECDI2030), a measurement tool intended to be used in household surveys to generate globally comparable population data on ECD outcomes. The development of the ECDI2030 involved several rounds of both quantitative and qualitative testing. This led to the identification of a set of questions which showed adequate psychometric properties [8]. These questions were considered appropriate for measurement across different languages as well as cultural, development and socioeconomic contexts when tested through several rounds of cognitive testing [3]. The ECDI2030 is comprised of 20 items which are administered to the mothers or primary caregivers of children about key milestones in the domains of health, learning and psychosocial well-being. Each item in the ECDI2030 captures specific developmental constructs nested within these three domains. These generate a single summary score reflecting the interlinkages among these domains [14]. Given that the application of the ECDI2030 generates data that countries can use for official reporting on SDG indicator 4.2.1, it is necessary to define criteria (standards) in order to transform the summative score obtained from the 20 items into a performance standard for classifying children as ‘developmentally on track’.

In the psychometric literature, establishing performance standards (also referred to as cut-scores) is accomplished through standard setting, whereby experts provide judgments as to which scores on a measure or test are indicative of different performance levels or categories [4]. Two main approaches are norm-referenced standards or criterion-referenced standards [1]. Norm-referenced standards are used when the focus is on interpreting test scores relative to the performance of others. Norm-referenced interpretations are common in medical and health applications, and require a sufficient amount of data, collected in a standardized way, that represents the breadth and depth of the intended population. This includes creating norms for measuring growth in children (in relation to age) as well as previous efforts to develop normed tools of ECD across countries [6]—but not with a measure with so few items as the ECDI2030.

Criterion-referenced standards are used when the focus is on interpreting test scores relative to the achievement of milestones or benchmarks. The process of creating criterion-referenced standards involves gathering judgments from a panel of subject matter experts (SMEs) who then establish a level of performance that indicates that a certain threshold or level of knowledge, skills, and abilities has been achieved [12]. The collection of these judgments (across items and/or across SMEs) is then used to establish a cut-score against which scores on a test or measure are interpreted. The judgment process and establishing the final cut-score are informed by empirical information and iterative discussions to assess the impact of a particular cut-score. The objective of the iterative process is to ensure that each SME is satisfied with her or his own item predictions and predicted cutoff scores. These are typically used with tests measuring knowledge and skills of students (e.g., [5, 12]). Without sufficient data from nationally representative samples, this can be a way of “benchmarking.”

A critical step of setting criterion-referenced cut-scores is developing an understanding of what differentiates each performance level from the one below at a *transition point*. For the ECDI2030, the transition point is identified by the developmental milestones that indicate a child of a given age (24, 36, or 48 months) is developmentally on track. For the purposes of this exercise, a child at this transition point is referred to as the ‘minimally on track child’ (MOTC).

The aim of this study was to establish criterion-referenced standards for the ECDI2030 based on informed judgements from subject matter experts in order to classify children as developmentally ‘on track’ or ‘not on track’.

## Methods

### ECDI2030 draft item set

We identified a set of 36 items that could potentially form the final ECDI2030 following a number of stages of item pool screening informed by results from four rounds of cognitive testing and dedicated field tests in three countries. This included 20 items within the learning domain, 11 items in the psychosocial well-being domain, and 5 items in the health domain. Details on the process for item selection are documented elsewhere (see [14]). The standard setting was implemented with this larger bank of 36 candidate items since the exercise preceded selection of the final item set.

**Fig. 1:**
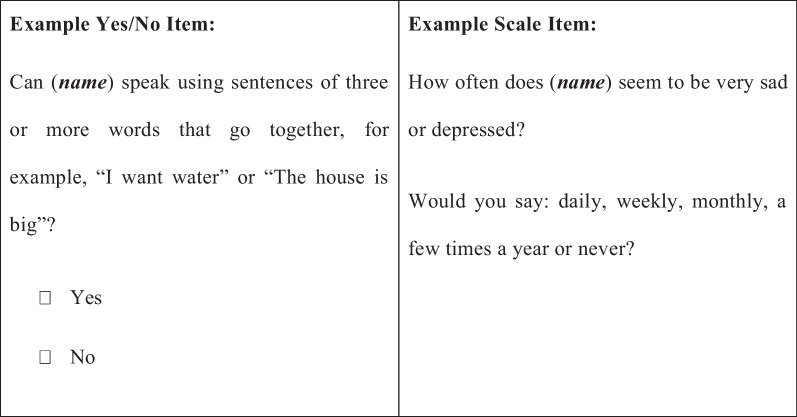
Example ECDI2030 items

Each item in the ECDI2030 is presented to a caregiver who is asked to indicate whether his or her child exhibits a specific behavior (for yes/no items) or how frequently the child exhibits the behavior (for scale items). Examples of each item type are included in Fig. 1.

### Standard setting

The Angoff [2] method, and its variations, are commonly employed for establishing criterion-referenced cut-scores. It requires subject matter experts (SMEs) to make judgments about each item on a test or measure against an established set of expectations for the performance level [12], such as being on track, for example. Calibrating these expectations among SMEs is paramount to successfully implementing the method. However, this is especially complex within the field of ECD, given that SMEs have expectations of children’s development that vary according to factors such as their own cultural frame of reference, profession, language, geographic region and consideration of children’s other environmental exposures such as participation in early childhood education.

The process typically followed for standard setting is to task SMEs with reviewing each item and then determine the knowledge or skills a child must have to be able to demonstrate/perform the item correctly. From this, the SME must then make a judgment as to how a subject (child) will likely perform at a minimum threshold for different performance levels—which in this case, is age. The recommended cut-score(s) for age is/are determined by combining these item-level judgments across the entire measure for each SME. There are two common variations of the Angoff method. In the “modified” Angoff, SMEs indicate the likelihood that a child at each performance level (age) would answer the item correctly [12]. With the “Yes/No” Angoff method [10], the task is simplified so that SMEs again think about whether a child will likely perform the item correctly at a certain age and simply indicate either “Yes” or “No” for each age range provided.

#### Pilot standard setting exercise

Given the potential benefits of implementing the Angoff method for standard setting, a pilot study was conducted to evaluate the feasibility of applying each variation described above to the ECDI2030 prior to the planned global standard setting. In the pilot, six experts (listed in Additional file 1: Annex A) met in Mexico for 1.5 days and practised this methodology using items previously discarded from the draft item set for the ECDI2030. These experts were asked to systematically apply the description of a child who is on the threshold for being developmentally on track, considering how expectations of a developmentally on track child would translate to performance on an item. The panel completed multiple rounds of standard setting, applying both rating strategies, with feedback information (in the form of data on the percentage of children who would be identified as on track and not on track according to their cut-scores using field test data from Mexico) being presented in between rounds to inform their subsequent judgments.

### Main standard setting exercise

#### Selection of members for the global panel

We identified a panel of fifteen global subject matter experts based on nominations from the Inter-agency and Expert Group on ECD Measurement (IAEG-ECD). The final set of experts was purposively selected to represent a range of expertise in early childhood development/developmental psychology/neurodevelopmental pediatrics. All those on the panel were senior professionals who had at least 10 years of practical expertise in conducting standardized assessments of children under age 5 in primary or tertiary health care, clinical, research and/or educational settings. We aimed to have collective representation across continents, cultures and languages as well as across psychology, education and medicine.

#### Procedure

##### Gaining a shared understanding

We provided panelists with background information on the development of the ECDI2030, the purpose and process for setting standards and their role as a SME in the process. Next, training was conducted by engaging the panelists in a “shared understanding” about what it means for a child to be considered developmentally on track at each age. Because the ECDI2030 covers three ages (i.e., 24, 36 and 48-month-old children), three descriptions for the ‘minimally on track child’ (MOTC) were developed. The panel was asked to brainstorm and discuss their expectations for what it means to be minimally on track in each of the three domains covered by the ECDI2030 (health, learning and psychosocial well-being). They began this discussion focusing on children aged 36 months and then repeated the process for children aged 24 months and then children aged 48 months. The development of these descriptions was not intended to force agreement upon the expert panel but rather to ensure they were all starting their judgmental process from the same point of reference.

##### Setting standards (Round 1)

Panelists were then provided training as to how they should translate the expectations captured in the descriptions into standard setting judgments, informed by their own professional practice and experience. Specifically, they were instructed to:

1. Review each ECDI2030 item and identify the behavior or skill being assessed
2. Determine if the behavior or skill being assessed is an expectation for a child who is aged 24 months and on track
3. Answer the key question—if you asked 100 mothers/caregivers of children aged 24 months who were minimally on track in their development:
  How many of them would endorse the item? *This applied to items that required a “Yes” or “No” response*
  How many of them (out of 100) would mark each response option? *This applied to the multiple-choice items that had more than two response options*
4. Repeat steps 2 and 3 for the MOTC aged 36 and 48 months

Panelists had the opportunity to practice their judgments on a few items and discuss their expectations and rationale within the group. The purpose of this discussion was not to come to consensus, but rather to allow panelists to hear how each other was translating the conceptual expectations for the MOTC into performance expectations on the ECDI2030.

After the training and practice, SMEs made their first round of standard setting ratings individually. Each SME used a tablet with a pre-loaded automatized form to register their ratings. The ratings involved making judgments about the 36 items and the expectations for MOTCs of each of the three age groups.

##### Setting standards (Round 2 with use of impact data)

Next, panelists were provided feedback on their first round of judgments including their individually recommended cut-scores as well as the group’s recommended cut-scores (mean, median, range). Additionally, as is common practice in standard setting [4], panelists were also presented with empirical information on the percentage of children who would be identified as on track for each age group by applying the group’s recommended cut-scores to some data. These data are referred to as ‘impact data’. In our case, we utilized impact data from field testing exercises which had been carried out by National Statistical Authorities in Mexico and in the State of Palestine on a larger bank of 58 items. These data were collected in 2018 and 2019, respectively, from representative and probabilistic samples of children aged 2–4 years based on responses provided by mothers/caregivers [14].

We encouraged the panel to discuss this impact data as well as some of those items that showed greater disparity in ratings, and others with a high degree of agreement. This discussion helped panelists evaluate how their judgmental process compared to the rest of the panel and helped the facilitators to evaluate the extent to which panelists were anchoring their judgments on a common understanding of the MOTC.

After the conclusion of this discussion, panelists worked independently to complete a second round of ratings. The purpose of this second round was to allow panelists to incorporate the feedback from the first round and any perspective gained during the discussion of the first round of results, and presentation of the impact data, into their final judgmental process. After the exercise was completed, panelists submitted an evaluation of the process and their confidence in the judgments they made.

#### Calculation of cut-scores

We calculated cut-scores by multiplying each panelist’s rating by the point value associated with the item/response and then summing the total values. ECDI2030 Yes/No items are scored as either 0 (for a “No” response) or 1 (for a “Yes” response). For these types of items, ratings were multiplied by 1. For example, if a panelist indicated that 40% of mothers/caregivers of MOTC aged 24 months would endorse an item, this value (40%) would be multiplied by 1 for a result of 0.40.

We chose to score ECDI2030 scale items as 1 for the response indicating the maximum level for exhibiting the behavior/skill and 0 for exhibiting the lowest level of the behavior/skill. We aimed to score the middle values as partial credit (e.g., 0.50) with the exact value varying based on the item and the number of response options. In order to determine scoring, SMEs made a judgment for each response option and then these values were multiplied by the assigned point value. The ratings for each response option (which sum to 1.0 or 100%) are multiplied by their respective point value and summed to determine the expected overall score for the MOTC for this item. Table 1 shows an example of how a score would be calculated if there were three response options. In this example, the panelist expected 30% of mothers/caregivers to respond ‘always’ to the item, 50% to respond ‘sometimes,’ and 20% to respond ‘never.’

**Table 1:** Sample calculation for scale item

Response	Point value	Rating	Score
Always	1	0.3	0.30
Sometimes	0.5	0.5	0.25
Never	0	0.2	0
Score on this item			0.55

#### Summary analyses to create cut-scores

For each round and each age, the panel’s mean, median, standard deviation and range were calculated. The mean is the mathematical average of all panelists’ recommendations for that particular age. The median value is the middle value of the recommendations across the entire panel. Differences between the mean and the median indicate that one or more recommendations was an outlier (i.e., very different from the group). The standard error (SE) of the mean is a measure of variability in the ratings among the group and the range represents the average recommended cut-score plus or minus two standard errors.

#### Calibration procedure of the newly created cut-scores on the 20 item ECDI2030

Calibration is a process used to transform scores from one test form to another form so that the transformed scores can be comparable [9]. The two forms are assumed to differ in scores by a constant value along the scale. As mentioned previously, the standard setting exercise was carried out based on a draft set of 36 candidate items that was further refined to a set of 20 items for the final version of the ECDI2030 [14]. However, only 18 of the 20 items in the final item set had actually been included in the standard setting exercise. Two items were not evaluated as part of the standard setting but were included on the final measure at a later stage (one on whether the child gets along well with other children and the other on the frequency with which the child seems to be very sad or depressed). Therefore, scores on the 18 items included in the standard setting were calibrated with that of the final 20 items and cut-scores were determined on the basis of the final measure.

## Results

The main recommendation from the pilot impact study was to utilize the modified form of the Angoff method for the global standard setting exercise. Although the experts in the Mexico pilot generally preferred the simpler ‘yes/no’ version of the form, the majority of the six experts felt that the probabilities generated more consistent results, especially given the need to set several age-specific cut-scores.

We convened fifteen experts (listed in Additional file 1: Annex A) from thirteen different countries (including low-, middle- and high-income) spanning five continents with geographical, cultural and linguistic diversity. All had at least 10 years’ experience in clinical assessment of ECD and were mainly medical doctors (neurodevelopmental pediatricians) or developmental psychologists. Eleven panelists attended the meeting in person and four joined virtually for the training and discussion portions.

**Fig. 2:**
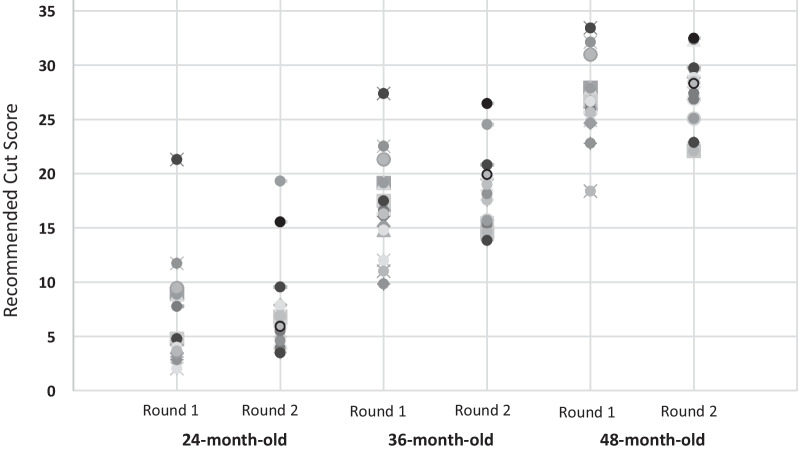
Subject Matter experts’ recommended cut-scores by age and round

The individual panelists’ recommendations for the entire set of items are shown in Fig. 2 by round. Each dot represents one panelist’s recommendation for that age (24, 36 and 48-month-olds on track) and round (round 1, round 2).

The recommendations from the entire panel are summarized in Table 2. For example, in Round 1, the panel’s recommended mean cut-score for a 24-month-old who is on track was 7.0 items out of 36.

**Table 2:** Standard setting results by round

	Mean	Median	SE	Range
*Round 1*				
24 months	7.0	4.7	1.4	4.3–9.7
36 months	17.1	16.4	1.3	14.5–19.6
48 months	26.7	26.6	1.0	24.7–28.8
*Round 2*				
24 months	7.7	6.8	1.1	5.5–10
36 months	18.4	18.1	0.9	16.5–20.2
48 months	28.0	28.5	0.8	26.4–29.5

In Round 1, the variation among the recommendations was greater as indicated by the higher standard errors and larger spread (Fig. 2). This observed variability in scores was expected as initial SME ratings were based on their expert opinion and results of the MOTC discussions. In Round 2, SMEs had more information on which to refine their judgments including group results from Round 1, impact data, and a panel discussion of results allowing for further reflection. Variability was less obvious in the second round with median recommendations (see Table 2) increasing slightly between rounds and variability (standard error, range, spread in ratings shown in Fig. 2) decreasing.

**Fig. 3:**
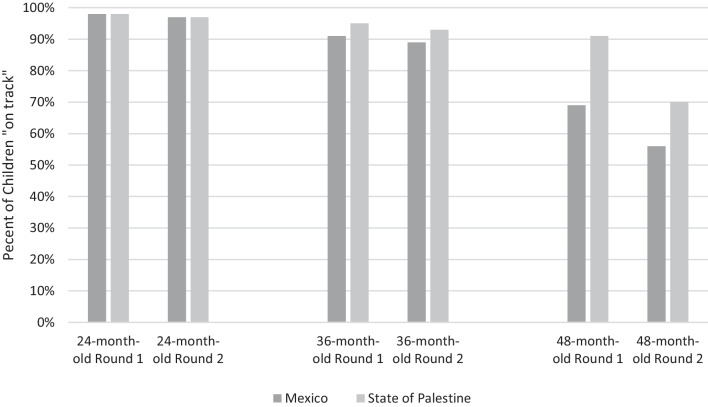
Proportion of children developmentally on track according to cut-scores for Round 1 and Round 2 in Mexico and State of Palestine, by age

The group’s median cut-scores for each age and round were applied to the field test data collected in Mexico and State of Palestine to calculate the proportion of children considered to be on track (Fig. 3). Based on the recommended standard created by the panel for children aged 24 months, there was a high percentage of children identified as on track in both Mexico and State of Palestine in both rounds (98% in Round 1 and 97% in Round 2). For children aged 36 months the panel’s cut-scores meant that more children were identified as on track in the State of Palestine (4% points higher in both rounds) than in Mexico. Finally, the proportion of children aged 48 months identified as on track (by applying the panel’s cut-score) was lower than the other two ages with more notable differences between the two countries (22% point difference between Mexico and State of Palestine in Round 1 and 14% point difference in Round 2).

### Calibrating the standard setting results to the final ECDI2030

The total cut-scores for each age were re-estimated using only the set of 18 items that were part of the standard setting and included on the final ECDI2030 (Table 3). The calibration was applied to determine the difference in difficulty between the set of 18 items and the final 20 item ECDI2030 with this relationship being used to obtain the standard setting recommendations for the final 20 item ECDI2030. As shown in Table 3, the cut-scores for the 20 item form were about 1 point higher than those for the 18 item form as a result of the mean calibration.

**Table 3:** Standard setting results for the 18 item form and the calibrated results for the final ECDI2030

	Min	Max	Mean	Median	SE	Range
*Original standard setting results (18 items)*						
24 months	1.8	9.7	3.7	3.3	0.5	2.7–4.8
36 months	7.2	12.3	9.0	8.6	0.4	8.2–9.8
48 months	11.5	16.1	14.0	14.5	0.3	13.3–14.7
*Calibrated standard setting results (20 items)*						
24 months	3.6	11.5	5.6	5.1	0.5	4.5–6.6
36 months	9.1	14.1	10.9	10.5	0.4	10–11.7
48 months	13.4	18.0	15.9	16.4	0.3	15.2–16.6

### Identification of the final performance standards for the ECDI2030

The final step in identifying the performance standards for the ECDI2030 involved establishing the final cut-scores to identify children developmentally on track. To inform this decision, field test data were used again to generate performance profiles according to different expectations of children’s performance based on the average calibrated cut-scores. The proportion of developmentally on track children rendered by each performance profile was reviewed for each country, taking into account the underlying expectation about children’s performance in general, and against a number of key disaggregation variables, such as sex and age, as well as exposure to poverty and other contextual factors such as household wealth, attendance in early childhood education and aspects of the home environment. The identification of the final cut-scores was guided by two additional considerations which were discussed in light of the field test results:

1. The need to impose an additional requirement on the minimum number of items within each domain: The idea of requiring a minimum number of items within each domain was to ensure that children were balanced in their development across domains and could not be classified as on track if they only demonstrated proficiency in one domain. It was decided, however, that such additional criteria to the cut-scores was not necessary because content coverage is already a core attribute of the ECDI2030 and was addressed in several ways throughout the different stages of instrument development (from the consultation process to define core sub-domains and constructs to the item selection process and psychometric modeling). In addition, further exploration of the field test data suggested that there were very few children who were lacking proficiency within one domain but were still classified as on track overall. Therefore, it was determined that the application of a single cut-score for each age range best supported the idea of ECD as being holistic in nature.
2. The need to include intermediate cut-scores for children between the ages of 24 and 35 months and between the ages of 36 and 47 months: Given how quickly development occurs during these early ages, we recognized that many children in these age groups may be identified as on track if they were in the latter part of the year (e.g., 32-month-old judged against the expectations for a 24-month-old). Therefore, the panel recommended the use of cut-scores by 6-month age groups for younger children (i.e., 24–29 months, 30–35 months, 36–41 months), which have also been applied and validated on other assessments related to ECD (see, for example [13]).

Discussion about these two considerations informed the calibration of the final set of standards from within the ranges recommended by the global panel (Table 3). For the 24-month-old standard, the upper end of the recommended range (6.6 rounded to 7) was selected on the basis of the estimated impact from the field test data. Similarly, for the 48-month-old standard, the lower end of the recommended range (15.2 rounded to 15) was chosen based on the estimated impact. Finally, the intermediate performance standards were identified as the median score within the range (i.e., the difference between the performance standard at the start of an age level and the performance standard for the next age level). Thus defined, the final age-specific cut-scores adopted to identify children developmentally on track were:

- 24–29 months: 7 of the 20 items
- 30–35 months: 9 of the 20 items
- 36–41 months: 11 of the 20 items
- 42–47 months: 13 of the 20 items
- 48–59 months: 15 of the 20 items

**Fig. 4:**
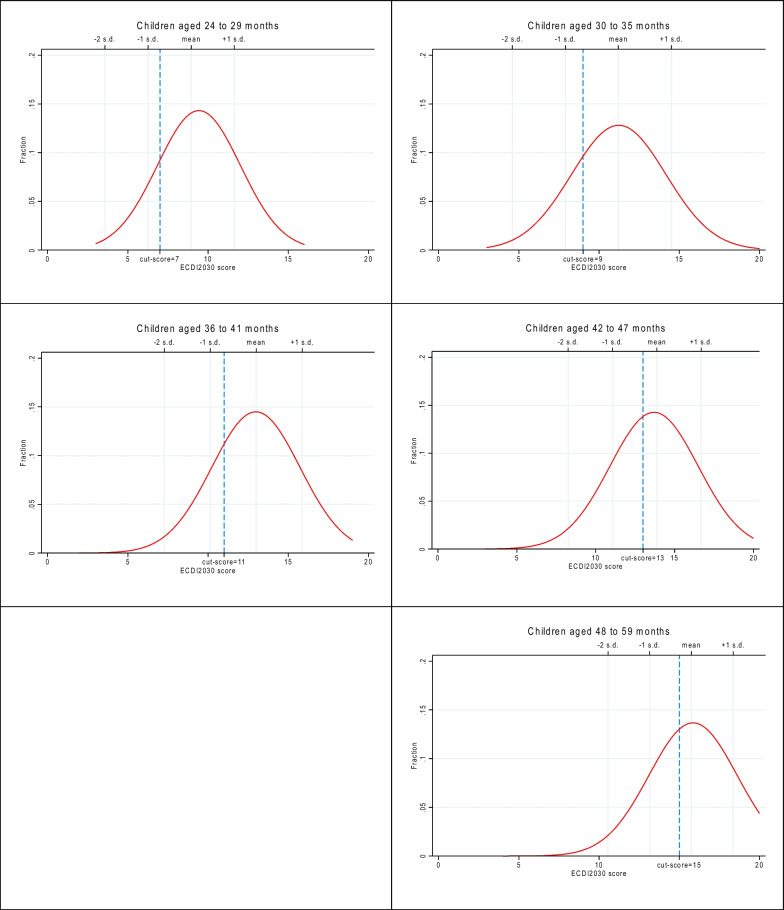
ECDI2030 score distribution and age-specific cut-scores, Mexico

**Fig. 5:**
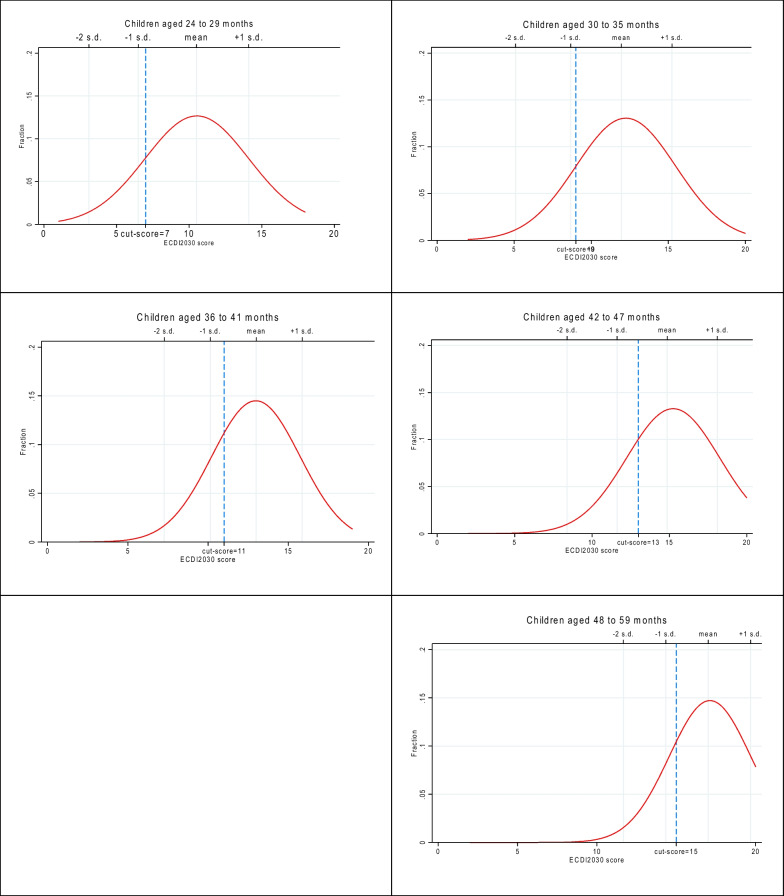
ECDI2030 score distribution and age-specific cut-scores, State of Palestine

Figures 4 and 5 show the impact results by applying these final cut-scores to the data from the Mexico and State of Palestine field tests, respectively, to compare the final cut-scores for each age group with the corresponding distributions, mean and standard deviations for the ECDI2030 score for the same age group in each country. Results show that the expectations for a “minimally on track child” generated by the five cut-scores requires a child’s performance on the ECDI2030 to fall somewhere between the mean and minus one standard deviation for the child’s age group.

Table 4 shows the percentage of children who were identified as developmentally on track by applying the final cut-scores to Mexico and State of Palestine data. The proportion of children identified as on track was higher in the State of Palestine than in Mexico. In both Mexico and State of Palestine, examination of the confidence intervals revealed that there was a higher proportion of girls, children living in the richest households and children of mothers with highest education levels classified as being developmentally on track. Higher proportions were also observed for children attending early childhood education, those with access to children’s books and those who engage in early stimulation activities with adults in the household.

**Table 4:** Percentage of children aged 24–59 months who are developmentally on track in Mexico and State of Palestine

	Mexico		State of Palestine	
	%	IC95%	%	IC95%
Children aged 24–59 months who are developmentally on track	74.6	70.7–78.2	84.3	80.8–87.2
Boys	72.2	66.5–76.4	79.8	74.3–84.4
Girls	80.6	74.4–84.9	89.2	84.8–92.5
Children living in the 20% poorest households	71.4	66.1–76.2	80.8	74.7–85.7
Children living in the 20% richest households	80.1	77.4–85.0	91.3	83.5–95.6
Children not attending early childhood education^a^	68.1	58.6–76-2	80.6	74.5–85.6
Children attending early childhood education^a^	72.4	67.3–77.0	91.8	84.9–94.6
No children's books in the household	66.1	58.6–72.9	79.2	74.3–83.3
At least three children's books in the household	81.0	75.1–85.1	95.2	91.4–97.2
Children who do not receive early stimulation	56.5	49.3–63.4	76.2	64.2–85.1
Children who receive early stimulation	80.1	76.3–83.4	85.4	81.7–88.4

^a^Children aged 36–59 months

## Discussion

Through a process of standard setting, we have generated performance standards for the ECDI2030 by establishing five age-specific cut-scores to identify children as developmentally on track. An important strength of the ECDI2030 as a measure is that it can be integrated into existing national data collection efforts to collect standard and internationally comparable data on ECD outcomes at the population level [14]. The measure is a public good and freely accessible and has been translated into a number of languages. It is accompanied by standard guidance and a set of implementation tools that include interviewer guidelines, customization and translation guidelines, training materials, syntaxes, tabulation plans and templates for reporting.

In the absence of sufficient data from nationally representative samples for a large number of diverse countries that could be used to create norm-referenced standards, we chose an established approach of setting criterion-referenced standards (the modified Angoff method, [2]) that has been widely applied to measurement tools in the fields of health and education to classify children as on track, taking into account impact data from pilot studies on the ECDI2030. One of the key strengths of our standard setting exercise is the fact that we identified a range of subject matter experts, all whom had extensive knowledge and expertise in the field of child development and who represented a wide range of geographic regions.

The use of our quantitative data (impact data generated by the field tests in Mexico and State of Palestine) as part of the standard setting enabled our process to be informed by meaningful data and clearly demonstrated the expected effects of education and wealth on ECD outcomes documented previously [7]. The application of the recommended cut-scores to the impact data did suggest that fewer children were identified as on track among older age groups in comparison with younger ages. Considering that development may be incrementally impacted by environmental and contextual factors such as attendance to early childhood education and school and household wealth as children get older [11] and that drivers to promote these may be less pronounced in some settings, these findings seem reasonable and fit well.

Our process of conducting more than one round of standard setting meant that standard errors decreased between rounds indicating that the panelists increased in their shared understanding of expectations as they discussed the results and their ratings. Our study demonstrated how panelists were able to differentiate expectations for children’s development at different ages, confirming that the ECDI2030 can measure children’s behaviors and skills relevant for different age groups. For a tool that will be used so widely, it was vital that we undertook work to establish performance standards for children at different ages. This is absolutely necessary as attainment of developmental milestones in the early years of life is intimately linked with age. We ensured that SMEs generated separate ratings by age for each item and then selected a final set of five age-specific cut-scores instead of the three cut-scores (for ages 24, 36 and 59 months) that were originally intended. We felt that this provided a better reflection of the progression and pace at which development happens among young children.

We acknowledge that the process by which performance standards were established for the ECDI2030 are not without some limitations. The ECDI2030 is designed and has been validated for population-level monitoring of ECD but is not appropriate for use as an individual-level assessment or as a developmental screening tool which require a different set of tools, conditions and frequency of administration. It is clear that the ability of the SMEs to generate perceived cut-scores was inherently limited to the set of items provided to them. Furthermore, some items within the tool do not discriminate as well by age (such as those within the psychosocial well-being domain), and therefore presented a greater challenge for defining performance standards.

At the stage of setting these standards, we did not have adequate empirical data from a sufficiently large enough sample of children across many countries to create norm-referenced standards. However, since its release in 2020, the ECDI2030 has been collected as part of nationally representative household surveys in as many as 25 countries. With the launch of the seventh round of the UNICEF-supported Multiple Indicator Cluster Surveys (MICS7) in 2023, which has fully integrated the ECDI2030, data could be generated for dozens of additional countries over the next few years. With this in mind, the availability of data on the ECDI2030 from such a large and diverse set of countries will enable the possibility of establishing age-specific norms on the basis of comparable empirical evidence that could then be compared to the criterion-referenced standards determined by the standard setting exercise. This will, in future, allow us to assess whether the existing cut-scores remain reasonable and valid or if they need to be revised in light of the expanded evidence base.

## Conclusion

This paper described the process of establishing performance standards for child development through the Angoff method for standard setting. It highlighted how the standard setting methodology, typically applied to measures in the health and education fields, could be applied to a measure of child development (the ECDI2030). By creating robust criterion-referenced standards, we have been able to ensure that the cut-scores related to age for the ECDI2030 are based on performance standards set by global experts in the ECD field for defining on and off track development.

### Supplementary Information

**Additional file 1.** Annex A.

## Data Availability

The datasets used and/or analyzed during the current study are available from the corresponding author on reasonable request.
