# Supplementary material for: Establishing performance standards for child development: learnings from the ECDI2030
Source: J Health Popul Nutr. 2023 Dec 12;42:140. doi: 10.1186/s41043-023-00483-2 (PMC10717755; doi:10.1186/s41043-023-00483-2)
Supplement: Supplementary file 1 — Additional file 1. Annex A. [file 41043_2023_483_MOESM1_ESM.docx]

**Additional file 1: Annex A**

**Global Panel Experts**

- Claudia Regina Lindgren Alves, Associated Professor, Department of Pediatrics, Universidade Federal de Minas Gerais/School of Medicine, Minas Gerais, Brazil
- Yvonne Becher, Chief Executive/ Director, Programme Development and Learning, The Child Development Centre, Hong Kong
- Maureen Black, Professor, University of Maryland School of Medicine & RTI International, Baltimore, USA
- Jennifer Grisham-Brown, Professor and Faculty Director of Early Childhood Laboratory, University of Kentucky, Lexington, USA
- Gauri Divan, Director, Child Development Group, Sangath, Delhi, India
- Kirsten Donald, Senior Specialist, Division of Developmental Pediatrics, Red Cross War Memorial Children's Hospital and Deputy Director, Neuroscience Institute, University of Cape Town, Cape Town, South Africa
- Melissa Gladstone, Senior Lecturer in Neurodevelopmental Pediatrics and International Child Health, University of Liverpool, Liverpool, United Kingdom
- Frances Page Glascoe, Professor of Pediatrics, Vanderbilt University,
  Nashville, USA
- Meta van den Heuvel, MD, PhD, Hospital for Sick Children, Toronto, Canada
- Gwendoline Kandawasvika, Senior Consultant, Primary Health Sciences Department, Faculty of Medicine and Health Sciences, University of Zimbabwe, Harare, Zimbabwe
- Shazia Maqbool, Professor & Chair Developmental-Behavioral Pediatrics Department, The Children's Hospital & Institute of Child Health, Lahore, Pakistan
- Fahmida Tofail, Scientist & Senior Consultant Physician, International Centre for Diarrhoeal Disease Research, Dhakka, Bangladesh
- Erika Marcela Osorio Valencia, Head of the Department of Developmental Neurobiology, National Institute of Perinatology, Mexico City, Mexico
- Tao Xin, Deputy Director of the National Assessment Center for Education Quality, Ministry of Education, Beijing, China
- Pia Zeinoun, (former) Assistant Professor, American University of Beirut, Beirut, Lebanon

**Pilot panel experts**

- María del Carmen Hernández Chávez, Researcher, National Institute of Perinatology, Mexico City, Mexico
- Diana Jazmín Virgen González, Jurisdictional Coordinator of Mental Health, Jalisco State Ministry of Health, Guadalajara, Mexico
- Guillermo Vargas López, Medical Doctor, Evidence Based Medicine Research Unit, Hospital Infantil de México, Mexico City, Mexico
- Cynthia Montalvo Rivera, Psychologist, Coahuila State Ministry of Education, Saltillo, Mexico
- Liliana Guadalupe Martell Valdez, Research Department Chief, Health Care Department, Coahuila State Ministry of Health, Saltillo, Mexico
- Erika Marcela Osorio Valencia, Head of the Department of Developmental Neurobiology, National Institute of Perinatology, Mexico City, Mexico
